# Reduction of Cytochrome *c* Oxidase During Vasovagal Hypoxia-Ischemia in Human Adult Brain: A Case Study

**DOI:** 10.1007/978-1-4614-7411-1_4

**Published:** 2013-03-25

**Authors:** Arnab Ghosh, Christina Kolyva, Ilias Tachtsidis, David Highton, Clare E. Elwell, Martin Smith

**Affiliations:** 00041 Neurocritical Care Unit, University College Hospitals, WC1N 3BG London, UK; 00042 Medical Physics & Bioengineering, University College London, WC1E 6BT London, UK

**Keywords:** Conscious Level, Moderate Hypoxia, Artery Flow Velocity, Discrete Wavelength, Middle Cerebral Artery Blood Flow

## Abstract

Near-infrared spectroscopy (NIRS)-derived measurement of oxidized cytochrome *c* oxidase concentration ([oxCCO]) has been used as an assessment of the adequacy of cerebral oxygen delivery. We report a case in which a reduction in conscious level was associated with a reduction in [oxCCO]. Hypoxaemia was induced in a 31-year-old, healthy male subject as part of an ongoing clinical study. Midway through the hypoxaemic challenge, the subject experienced an unexpected vasovagal event with bradycardia, hypotension and reduced cerebral blood flow (middle cerebral artery blood flow velocity decrease from 70 to 30 cm s^−1^) that induced a brief reduction in conscious level. An associated decrease in [oxCCO] was observed at 35 mm (−1.6 μM) but only minimal change (−0.1 μM) at 20-mm source-detector separation. A change in optical scattering was observed, but path length remained unchanged. This unexpected physiological event provides an unusual example of a severe reduction in cerebral oxygen delivery and is the first report correlating change in clinical status with changes in [oxCCO].

## Introduction

Cytochrome *c* oxidase is the final electron acceptor in the mitochondrial electron transport chain, and its oxidation state, measured by NIRS as [oxCCO], has thus been proposed as a marker of the adequacy of cerebral oxygen delivery (DO_2_) [1]. However, prior studies of moderate hypoxia achieved only modest reductions in DO_2_, leaving the relationship between DO_2_ and ∆[oxCCO] unclear [2]. Furthermore, interpretation of [oxCCO] measurements is difficult as there are no adult human data regarding its total concentration or resting oxidation state.

We aimed to address some of these concerns with studies using a hybrid NIR spectrometer, the pHOS, to measure [oxCCO] and other optical parameters whilst modulating DO_2_ in a cohort of healthy volunteers [3]. We report the case of a vasovagal event that occurred during one such study and explore the insights into cerebral physiology gleaned from this unexpected event.

## Methods

A single case was selected from a larger group study. This study was approved by the Institutional Research Ethics Committee, and written informed consent was provided by the subject, a 31-year-old male who had been screened for pre-existing medical conditions, was selected as he suffered from vasovagal pre-syncope during a challenge that comprised induction of isocapnic hypoxia with a target arterial oxygen saturation (SpO_2_) of 80 % [3].

The pHOS, described in more detail elsewhere [4], combines frequency domain (FD) and broadband (BB) components. Changes in chromophore concentration were estimated by using the changes in light attenuation as measured by the BB spectrometer, using the UCLn algorithm to resolve for three chromophores – oxyhaemoglobin (HbO_2_), deoxyhaemoglobin (HHb) and oxCCO – between 780 and 900 nm. A fixed differential path length factor (DPF) of 6.26 [5] was used to enable comparison with previous studies. The FD component measured the absolute absorption and scattering coefficients (μa and μs, respectively) at discrete wavelengths 690, 750, 790 and 850 nm, allowing the estimation of DPF at each of these wavelengths using the diffusion approximation [6]. The pHOS optode was placed over the FP1 point on the right side of the forehead.

Other monitoring included beat-to-beat SpO_2_, continuous non-invasive arterial blood pressure, and transcranial Doppler (TCD) ultrasonography was used to measure middle cerebral artery flow velocity (Vmca), insonating through the right temporal window, ipsilateral to pHOS monitoring. Estimated relative cerebral oxygen delivery (ecDO_2_) was calculated as the product of changes in SpO_2_ and Vmca (relative to their initial values) [2]. Synchronization between the pHOS and other monitors was performed by means of a signal voltage output by the frequency domain spectrometer for the length of recording; all data were resampled to a sample period of 3.2 s across the length of recording. Twenty-second data windows were selected for the reporting of summary data. Changes in hemoglobin species concentration are expressed as total hemoglobin (∆[HbT] = ∆[HbO_2_] + ∆[HHb]) and hemoglobin difference (∆[HbDiff] = ∆[HbO_2_] − ∆[HHb]). All analysis was carried out in Matlab® 2011b.

## Results

**Fig. 4.1:**
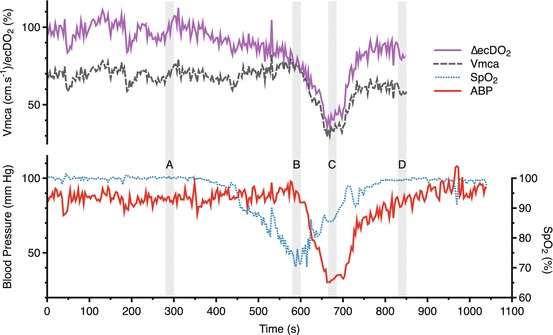
Changes in systemic and physiological parameters. Data averaging periods *A* baseline, *B* hypoxia, *C* hypoxia-ischemia, *D* recovery

Approximately 600 s after the commencement of recording, during the nadir of hypoxia, the subject suffered from sudden-onset bradycardia and hypotension (a typical vasovagal episode), resulting in a reduction in ecDO_2_ to 41.5 % of baseline values (Table 4.1 and Fig. 4.1). During this time, the subject became briefly unresponsive. When this occurred, he was laid flat and the breathing circuit replaced with a Mapleson C circuit delivering high-flow oxygen.

**Table 4.1:** Mean values of systemic and cerebral physiological parameters observed during four observed phases

		Baseline	Hypoxia	Hypoxia-ischemia	Recovery
**ABP (mmHg)**		85.4	88.9	32.1	83.5
**HR**		78.0	117	41.3	101
**Vmca (cm s** ^**−1**^ **)**		70.4	73.9	34.1	57.8
**SpO**_**2**_ **(%)**		100	72.6	86.3	99.4
**ecDO**_**2**_ **(%)**		99.8	75.8	41.5	81.2
**∆[oxCCO] (μM)**	3.5 cm	0	−0.74	−1.56	−0.13
	3.0 cm	0	−0.38	−0.62	0.008
	2.5 cm	0	−0.30	−0.55	0.045
	2.0 cm	0	−0.14	−0.16	0.23

**Fig. 4.2:**
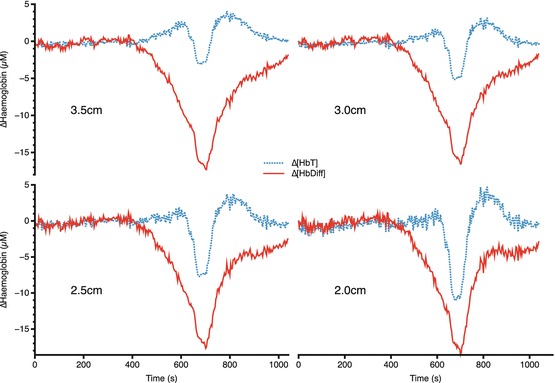
Changes in hemoglobin concentrations at four source-detector separations

**Fig. 4.3:**
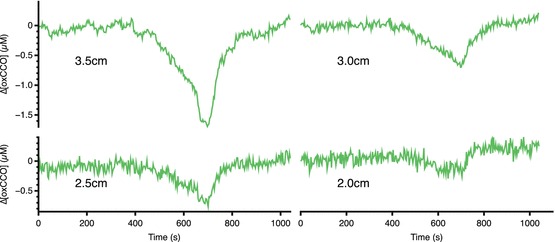
Changes in [oxCCO] at four source-detector separations

**Fig. 4.4:**
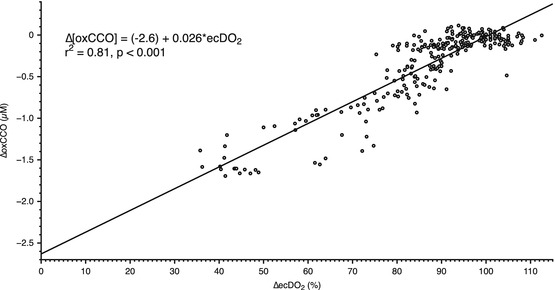
Relationship between ecDO_2_ and ∆[oxCCO] at 3.5 cm source-detector separation

**Fig. 4.5:**
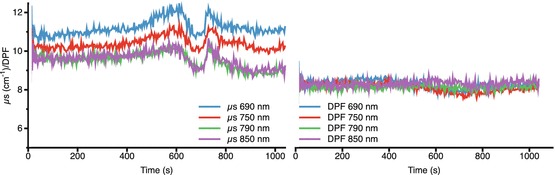
Changes in μs and DPF

Hemoglobin species showed a consistent pattern of change with a decrease in both [HbT] and [HbDiff] seen at all source-detector separations (Fig. 4.2). By contrast, [oxCCO] showed a source-detector separation-dependent decrease (∆[oxCCO] −1.6 μM at 3.5 cm c.f. −0.1 μM at 2.0-cm source-detector separation), with larger decreases seen at further source-detector separations (Fig. 4.3). Comparison of ∆[oxCCO] (observed at the 3.5-cm detector) with ∆ecDO_2_ suggests a linear relationship (Fig. 4.4) between the two variables (*r* ^2^ = 0.81, *p* < 0.001). There appeared to be a roughly 10 % decrease in the μs observed at all four discrete wavelengths, but this did not translate into an apparent change in DPF (Fig. 4.5).

## Discussion

We report a reduction in cranial [oxCCO] measured with the pHOS during a vasovagal pre-syncope in a healthy adult volunteer. This reduction was larger in further (3.5 cm) than closer (2.0 cm) source-detector separations. The pattern of [oxCCO] reduction has been related to neurological outcome following cardiopulmonary bypass [7], and asymptomatic reductions in [oxCCO] have been reported during moderate hypoxia [2], but this is the first change in [oxCCO] to be correlated to changes in conscious level and the largest change to be reported in humans.

Whilst hypoxia is a recognized cause of syncope, the occurrence of bradycardia and hypotension indicates a vasovagal etiology [8], in this case, provoked by – as the subject reported – the shock of seeing his reduced SpO_2_ reading. This vasovagal response resulted in a profound reduction in cerebral blood flow (observed by both NIRS and TCD), and this led to a significant reduction in DO_2_.

Prior experiments have achieved only modest reductions in DO_2_, leaving questions unanswered about the relationship between DO_2_ and ∆[oxCCO] [2]. In particular, it was unclear whether the relationship between DO_2_ and ∆[oxCCO] was linear or whether there was a DO_2_ threshold below which rapid CCO reduction occurs. However, our data (Fig. 4.4) suggest a linear relationship between ∆[oxCCO] and ∆ecDO_2_ (*r* ^2^ = 0.81, *p* < 0.001). From this, the extrapolated value of ∆[oxCCO] of −2.6 μM with zero cerebral oxygen delivery is suggestive of a resting oxidized CCO concentration of around 2.6 μM. Although no reduction of this size has been reported in humans – understandably as a reduction in DO_2_ to nothing is impractical in humans – these values are consistent with animal anoxia models [9, 10].

Given the distance dependence of the [oxCCO] but not hemoglobin changes, we consider spectral crosstalk to be unlikely. Similarly, the modest μs changes that were observed appeared consistent in their spectral expression across 690–850 nm and thus are unlikely to account for the [oxCCO] changes seen. DPF showed no significant qualitative change during the hypoxic or ischaemic phases of the study, although the absolute values were larger than those that were previously reported [5]; using the measured values of DPF rather than the fixed value of 6.26 would thus change the magnitude, but not the qualitative profile of our observed chromophore changes.

Whilst we are aware of the limitations of extrapolating from a single case report, these data, especially when considered in conjunction with the results of the larger cohort of (uncomplicated!) hypoxia and hypercapnia studies [3], further underline confidence in the ability to use NIRS to measure [oxCCO] as a marker of cellular energy status.
